# Enhanced Coexistence of Quantum Key Distribution and Classical Communication over Hollow-Core and Multi-Core Fibers

**DOI:** 10.3390/e26070601

**Published:** 2024-07-15

**Authors:** Weiwen Kong, Yongmei Sun, Tianqi Dou, Yuheng Xie, Zhenhua Li, Yaoxian Gao, Qi Zhao, Na Chen, Wenpeng Gao, Yuanchen Hao, Peizhe Han, Yang Liu, Jianjun Tang

**Affiliations:** 1 China Telecom Research Institute, Beijing 102200, China; kongww1@chinatelecom.cn (W.K.);; 2 The State Key Laboratory of Information Photonics and Optical Communications, School of Information and Communication Engineering, Beijing University of Posts and Telecommunications, Beijing 100876, China; gaoyaoxian@bupt.edu.cn

**Keywords:** quantum key distribution, hollow-core fiber, multi-core fiber, noise analysis, wavelength allocation

## Abstract

In this paper, we investigate the impact of classical optical communications in quantum key distribution (QKD) over hollow-core fiber (HCF), multi-core fiber (MCF) and single-core fiber (SCF) and propose wavelength allocation schemes to enhance QKD performance. Firstly, we theoretically analyze noise interference in QKD over HCF, MCF and SCF, such as spontaneous Raman scattering (SpRS) and four-wave mixing (FWM). To mitigate these noise types and optimize QKD performance, we propose a joint noise suppression wavelength allocation (JSWA) scheme. FWM noise suppression wavelength allocation and Raman noise suppression wavelength allocation are also proposed for comparison. The JSWA scheme indicates a significant enhancement in extending the simultaneous transmission distance of classical signals and QKD, reaching approximately 100 km in HCF and 165 km in MCF under a classical power per channel of 10 dBm. Therefore, MCF offers a longer secure transmission distance compared with HCF when classical signals and QKD coexist in the C-band. However, when classical signals are in the C-band and QKD operates in the O-band, the performance of QKD in HCF surpasses that in MCF. This research establishes technical foundations for the design and deployment of QKD optical networks.

## 1. Introduction

Quantum key distribution (QKD) leverages the fundamental principles of quantum mechanics to establish secure key exchanges between distant communication entities [1]. When integrated with one-time pad technology, it offers a theoretically secure foundation for symmetric key encryption systems. In recent advancements, QKD has demonstrated significant progress in both protocols and networks [2,3,4]. Notably, the secure key rate can reach 110 Mbps [5], and the longest transmission distance has extended to 1002 km when implemented using twin-field QKD [6]. These advancements have catalyzed the development of large-scale QKD networks.

The independent deployment of dedicated QKD networks presents challenges for large-scale implementation due to high fiber costs. Integrating QKD functionality into existing optical networks, encompassing metropolitan area networks [7], gigabit-capable passive optical networks [8], optical transport networks [9] and ethernet passive optical networks [10], offers a more cost-effective and practical approach to deployment. While co-transmission of classical and quantum signals over single-core fiber (SCF) can mitigate fiber deployment expenses, it poses challenges to the performance of QKD due to noise induced by classical signals, such as spontaneous Raman scattering (SpRS) and four-wave mixing (FWM) noise [11,12]. Although reducing the power of classical signals can mitigate noise interference on QKD, it concurrently imposes higher sensitivity requirements on classical signal receivers, which adversely affects classical communications.

Fortunately, advancements in fiber technology offer promising opportunities for the simultaneous transmission of classical and quantum signals [13,14]. Hollow-core fiber (HCF) represents a departure from traditional doped high-purity silicon fiber cores. Its distinct advantage is that the fiber performance is not limited by the material properties of the core, which substantially reduces the nonlinearity effect and fiber attenuation [15,16]. Moreover, the current attenuation in HCF has been reduced from 0.174 dB/km [17] to below 0.11 dB/km [18], and the lowest can reach 0.05 dB/km, approaching its theoretical loss limit [14]. This significant reduction in attenuation levels holds promising implications for the simultaneous transmission of classical and quantum signals, offering promising prospects for integrated optical communication systems.

Although HCF has significant advantages in theory and research, its loss remains high in experimental environments, such as 1.3 dB/km [19] and more than 2 dB/km [20]. This high loss limits the transmission distance for quantum signals and increases costs. Meanwhile, the manufacturing process of HCF still needs to be further improved, and the current process can only support a limited length. However, with the development of and demand for HCF, as well as the advancement of loss and manufacturing technology, these challenges will gradually be addressed, and HCF still has a future.

Additionally, weakly coupled multi-core fiber (MCF) features multiple cores within a single cladding [21]. The spatial isolation between these cores, facilitated by the core pitches, effectively reduces noise interference from one core to another core [13]. Leveraging these characteristics, MCF demonstrates an augmented transmission capacity and heightened spatial efficiency. As a result, the simultaneous transmission of classical and quantum signals through HCF and MCF holds great promise for QKD optical networks.

Recently, researchers have explored the simultaneous transmission of classical and quantum signals in HCF and MCF [19,22,23,24,25,26,27]. In a pioneering effort, a 1.6 Tbps classical data signal and QKD were simultaneously transmitted over a 2 km of HCF, with a combined classical signal power totaling 0 dBm [19,28]. Subsequently, a coexistence experiment involving classical communication and QKD with a wavelength of 25 was successfully demonstrated over a 7.7 km HCF [22]. MCF was employed alongside QKD in a 53 km seven-core fiber, achieving a secure key rate of up to 605 kbps for the first time [23]. Subsequent experiments based on MCF revealed enhanced performance metrics, with the secure key rate peaking at 105.7 Mbps [29], the classical capability reaching 11.2 Tbps [30] and the attained tolerated power of the classical signal being 25 dBm [31]. Moreover, advanced resource allocation schemes have been investigated, focusing primarily on core and wavelength resources to further optimize the secure key rate [32,33]. Consequently, the coexistence capabilities of classical signals and QKD leveraging HCF and MCF exhibited substantial advantages in experiments.

Although experimental studies on co-transmission based on HCF and MCF have made initial progress, the characteristics of noise in these fibers and the varying impacts of different noises on QKD performance, particularly in HCF, have not been thoroughly investigated. Furthermore, research on resource allocation schemes tailored to the distribution characteristics of noise remains incomplete. It is essential to explore efficient resource allocation strategies to enhance QKD performance in co-transmission scenarios involving classical and quantum signals over HCF and MCF.

In this paper, we analyze the noise generated by classical signals on quantum channels over HCF and MCF. Subsequently, we propose a joint noise suppression wavelength allocation (JSWA) scheme to simultaneously suppress FWM noise and SpRS noise. An FWM noise suppression wavelength allocation scheme and Raman noise suppression wavelength allocation scheme are proposed to suppress FWM noise and SpRS noise, respectively, and are used as comparison schemes. The results show that the FWM noise in HCF was lower than that in MCF at most distances, and the SpRS noise in HCF was always higher than that in MCF. Furthermore, the proposed noise suppression scheme significantly improved the secure transmission distance. Moreover, when both classical and quantum signals were in the C-band, the coexisting classical signal power per channel could reach up to 17 dBm in HCF and exceed 30 dBm in MCF.

## 2. Theoretical Analysis in a Coexistence System

In this section, we first design a simultaneous transmission architecture for classical and quantum signals. Secondly, we theoretically analyze the noise affecting the quantum channels within this architecture. Subsequently, we propose wavelength allocation schemes aimed at noise suppression based on HCF, MCF and SCF. Finally, we analyze the theory for calculating the secure key rate under the influence of noise.

### 2.1. Coexistence System Architecture

**Figure 1:**
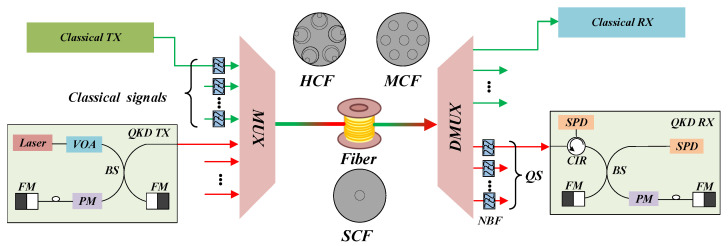
Simultaneous transmission architecture of classical signals and quantum signals over multiple fibers (VOA = variable optical attenuator; BS = beam splitter; PM = phase modulator; FM = Faraday mirror; MUX = multiplexer; DMUX = demultiplexer; HCF = hollow-core fiber; MCF = multi-core fiber; SCF = single-core fiber; QS = quantum signals; CIR = optical circulator; NBF = narrow-band filter; SPD = single-photon detector).

Figure 1 depicts the simultaneous transmission architecture for classical and quantum signals. Within this architecture, classical and quantum signals coexist in HCF, MCF or SCF.

The classical signal passes through the NBF to filter out the amplifier’s spontaneous emission noise and is subsequently multiplexed with the quantum signal through the MUX. In the QKD transmitter, the VOA adjusts the quantum signal intensity, and the BS, PM, and FM form a Faraday–Michelson interferometer. For the QKD receiver, the NBF filters out the out-of-band noise introduced by classical signals, and the SPD detects the photon.

### 2.2. Noise Analysis

In addition to the inherent dark count and channel crosstalk noise of the SPD, the primary noise sources affecting the quantum channel include FWM and SpRS noise generated by the classical signal. These noise types are in-band and resistant to conventional filtering methods. The subsequent analysis delves into the characteristics of FWM and SpRS noise over SCF, HCF, and MCF.

#### 2.2.1. Four-Wave Mixing Noise

FWM is a nonlinear optical phenomenon of interaction between two or three wavelengths in fiber which generates new frequencies [34,35]. FWM noise can occupy channels which overlap with the quantum channel, potentially interfering with the quantum signal. Assuming that the three frequencies of the classical signal are $f_{i}$, $f_{j}$ and $f_{k}$, the new frequency generated by FWM is expressed as [36]

(1) $$f_{ijk}=f_{i}+f_{j}-f_{k}\left(i,j\neq k\right).$$

The FWM noise power for SCF and HCF can be expressed as [19,36]

(2) $$\begin{array}{c} P_{FWM,s/h}\left(L\right)=\frac{\eta_{s/h}D^{2}\gamma_{s/h}^{2}P_{i}P_{j}P_{k}exp\left(-\alpha_{s/h}L\right)}{9\alpha_{s/h}^{2}}\cdot {\left[1-exp(-\alpha_{s/h}L)\right]}^{2}, \end{array}$$

where *D* is the degeneracy factor, $\gamma_{s/h}$ is the nonlinear coefficient of SCF and HCF and $\alpha_{s/h}$ denotes the attenuation of SCF and HCF, while $P_{i}P_{j}P_{k}$ is the power of three classical signals and $\eta_{s/h}$ is the FWM efficiency of the SCF and HCF, which can be expressed as

(3) $$\eta_{s/h}=\frac{\alpha_{s/h}^{2}}{\alpha_{s/h}^{2}+\Delta \beta_{s/h}^{2}}\left(1+\frac{4\exp\left(-\alpha_{s/h}L\right)\sin^{2}\left(\frac{\Delta \beta_{s/h}L}{2}\right)}{{\left[1-\exp\left(-\alpha_{s/h}L\right)\right]}^{2}}\right)$$

Here, $\Delta \beta_{s/h}$ is the phase matching factor, which can be expressed as [36]

(4) $$\Delta \beta_{s/h}=\frac{2\pi \lambda^{2}}{c}\left|f_{i}-f_{k}\right|\cdot \left|f_{j}-f_{k}\right|\left\{D_{c,s/h}+\frac{dD_{c,s/h}}{d\lambda }\left(\frac{\lambda^{2}}{2c}\right)\times \left[\left|f_{i}-f_{k}\right|+\left|f_{j}-f_{k}\right|\right]\right\}$$

where λ is the wavelength of the FWM light, *c* is the speed of light, $D_{c,s/h}$ is the fiber-chromatic dispersion coefficient and $\frac{dD_{c,s/h}}{d\lambda }$ is the dispersion slope.

The light-guiding mechanism of HCF differs from that of solid-core fiber, which leads to distinct nonlinear coefficients. The nonlinear coefficients of SCF and HCF can be expressed as [37]

(5) $$\gamma_{s/h}=\frac{2\pi n_{2,s/h}}{\lambda A_{eff,s/h}},$$

where $n_{2,s/h}$ is the Kerr nonlinear parameter of SCF and HCF, λ is the optical wavelength and $A_{eff,s/h}$ is the effective mode area of SCF and HCF.

For MCF, FWM noise is mainly generated by the interaction between intra-core FWM noise and inter-core crosstalk noise. The intra-core FWM noise power can be calculated using the method for SCF. The inter-core crosstalk (ICXT) noise is calculated as follows [38,39]:

(6) $$\begin{array}{cc} P_{ICXT} & =P\cdot exp\left(-h_{mn}L\right)sinh\left(h_{mn}L\right)exp\left(-\alpha_{m}L\right)\\ & =P\cdot ICXT, \end{array}$$

where *P* represents the classical signal power, $\alpha_{m}$ represents the attenuation of the MCF and $h_{mn}$ denotes the coupling coefficient of the MCF.

The FWM noise in MCF can be represented by [40]

(7) $$\begin{array}{cc} P_{FWM,m} & =P_{FWM\to ICXT}+P_{ICXT\to FWM}\\ & \approx P_{FWM,s}\cdot ICXT, \end{array}$$

where $P_{FWM\to ICXT}$ is the noise power in the quantum channels from the FWM noise through ICXT and $P_{ICXT\to FWM}$ is the noise power in the quantum channels from the ICXT noise through FWM. Here, $P_{FWM\to ICXT}$ can be disregarded due to its significantly weaker power compared with $P_{ICXT\to FWM}$.

#### 2.2.2. Spontaneous Raman Scattering Noise

SpRS noise arises from the interaction between optical signals within the fiber. SpRS noise in SCF and HCF can be expressed as [41]

(8) $$P_{SpRS,s/h}=P\cdot exp\left(-\alpha_{s/h}L\right)\cdot L\cdot \rho_{s/h}\left(\lambda_{c},\lambda_{q}\right),$$

where $\rho_{s/h}\left(\lambda_{c},\lambda_{q}\right)$ represents the Raman scattering factor for SCF and HCF, $\lambda_{c}$ denotes the wavelength of the classical signal and $\lambda_{q}$ signifies the wavelength of the quantum signal.

The SpRS noise in MCF can be represented by [40]

(9) $$\begin{array}{cc} P_{SpRS,m} & =P_{SpRS\to ICXT}+P_{ICXT\to SpRS}\\ & =2\cdot P_{SpRS,s}\cdot ICXT, \end{array}$$

where $P_{SpRS\to ICXT}$ represents the noise power on quantum channels originating from SpRS noise through ICXT and  $P_{ICXT\to SpRS}$ denotes the noise power in the quantum core stemming from ICXT noise through SpRS.

### 2.3. Proposed Noise Suppression Schemes

Based on noise analysis, to mitigate SpRS and FWM noise over HCF, MCF and SCF, we propose a joint noise suppression wavelength allocation (JSWA) scheme. Furthermore, an FWM noise suppression wavelength allocation (FSWA) scheme and Raman noise suppression wavelength allocation (RSWA) scheme are proposed for comparision.

**Figure 2:**
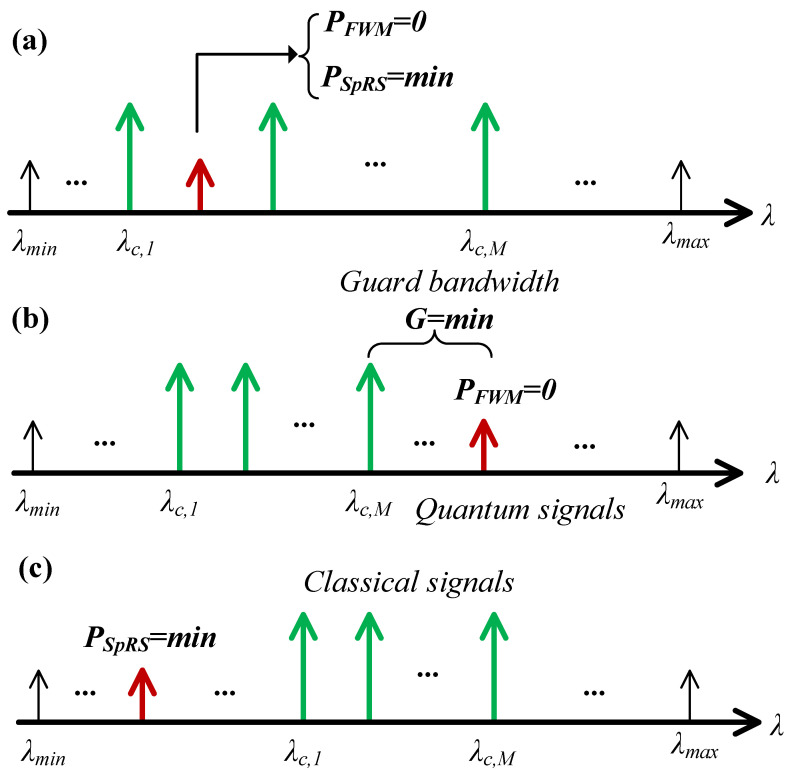
Wavelength allocation schemes: (**a**) JSWA; (**b**) FSWA; and (**c**) RSWA.

The scheme diagram is presented in Figure 2. Here, $\lambda_{c,1},\ldots ,\lambda_{c,M}$ denote the M classical wavelength, while $\left[\lambda_{min},\lambda_{max}\right]$ represents the candidate quantum wavelength range. The key concepts and implementation algorithms for the JSWA, FSWA and RSWA schemes are detailed as follows.

**JSWA scheme**: The JSWA scheme is designed to effectively suppress FWM noise and mitigate SpRS noise in the quantum channels. It employs an interleaving strategy for classical and quantum channels which not only completely avoids FWM noise but also reduces SpRS noise across all candidate quantum wavelengths. The channel interleaving strategy means allocating quantum channels between classical channels. Through the channel interleaving strategy, FWM noise generated by classical signals will not fall between classical channels, and the SpRS noise power near classical channels is usually low. Wavelengths without FWM noise and with minimal SpRS noise are prioritized as quantum wavelengths, as illustrated in Figure 2a.

The JSWA scheme is executed using Algorithm 1. Line 1 determines the wavelength range for the candidate quantum channels. Line 2 then calculates the FWM noise generated by M classical signals within this wavelength range. If the FWM noise power in a candidate quantum channel is zero, then the SpRS noise on that channel is subsequently calculated, and the corresponding wavelength and SpRS noise power are recorded, as implemented in lines 3–7. Lines 9 and 10 handle the sorting and selection process, identifying wavelengths with minimal SpRS noise power and designating them as quantum wavelengths.

**Algorithm 1:** JSWA scheme in HCF, MCF and SCF

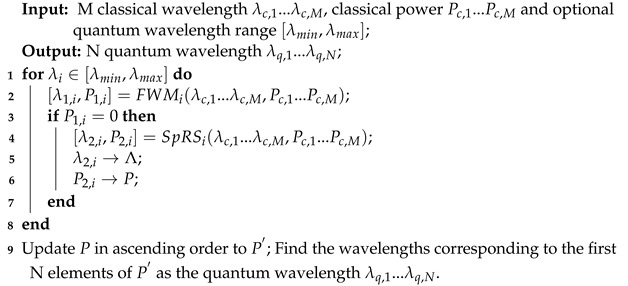



**FSWA scheme**: The primary objective of the FSWA scheme is to supress FWM noise in the quantum channels. Increasing the guard band G between quantum wavelengths and classical wavelengths is the first priority. Wavelengths without FWM noise can serve as candidate quantum wavelengths, as illustrated in Figure 2b. Quantum wavelengths which are proximate to the classical wavelengths among the candidate quantum wavelengths are prioritized for selection because this method occupies a small wavelength range, thereby improving resource utilization.

The FSWA scheme is implemented through Algorithm 2. Line 2 calculates the FWM noise power on the *i*th wavelength generated from M classical signals. When the FWM noise power on the *i*th wavelength is zero, the wavelength spacing between the *i*th wavelength and the classical wavelength is determined in Line 4. The wavelengths of the candidate quantum channels and their corresponding wavelength spacings with the classical wavelength are stored in arrays Λ and *D*, respectively, as shown in lines 5 and 6. Subsequently, the wavelength spacings are arranged in ascending order, and the candidate quantum wavelengths closest to the classical wavelengths are selected as the prioritized quantum wavelengths, as depicted in lines 9 and 10.

**RSWA scheme**: The RSWA scheme primarily aims to minimize SpRS noise in the quantum channels. It involves traversing the SpRS noise power across the candidate quantum wavelengths, prioritizing wavelengths with minimal SpRS noise for selection as quantum wavelengths, as illustrated in Figure 2c.

The RSWA scheme is executed using Algorithm 3. Line 2 calculates the SpRS noise power on the *i*th wavelength generated from M classical signals. The candidate quantum wavelengths and their corresponding SpRS power are stored in arrays Λ and *P*, respectively, as shown in lines 3 and 4. Lines 6 and 7 handle the sorting and selection process, identifying wavelengths with low SpRS noise power and designating them as quantum wavelengths.

The JSWA scheme can simultaneously suppress FWM noise and reduce SpRS noise. However, the close channel spacing between quantum and classical channels in this scheme demands high equipment isolation in practical engineering applications. Without sufficient isolation, channel crosstalk from classical signals can degrade QKD performance. In contrast, although the FSWA and RSWA schemes can only suppress either FWM noise or SpRS noise, the larger spacing between quantum and classical channels makes these schemes easier to implement in practical engineering scenarios.

**Algorithm 2:** FSWA scheme in HCF, MCF and SCF

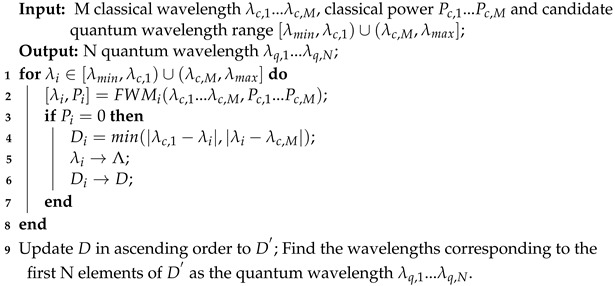



**Algorithm 3:** RSWA scheme in HCF, MCF and SCF

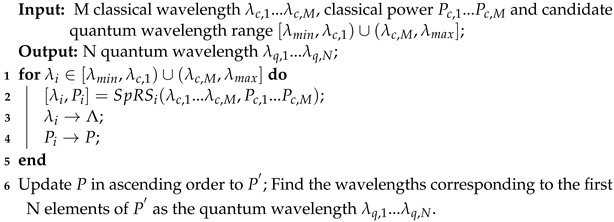



### 2.4. Secure Key Rate Analysis

The decoy state QKD protocol effectively addresses the photon number-splitting attack arising from multiple photons per pulse and is widely employed in experiments [5,42,43,44]. The lower bound $R_{pulse}$ of the secure key rate in the decoy state BB84 protocol is [45]

(10) $$R_{pulse}\geq q\left\{Q_{1}\left[1-H_{2}\left(E_{1}\right)\right]-Q_{\mu }fH_{2}\left(E_{\mu }\right)\right\},$$

where *q* is the protocol-related efficiency, which is 0.5 in the BB84 protocol, *f* is 1.15 when utilizing the low-density parity check code, $H_{2}\left(x\right)$ represents binary Shannon information entropy and $Q_{1}$, $E_{1}$, $Q_{\mu }$ and $E_{\mu }$ are the single photon gain, the error rate caused by the single photon state, the gain of the signal state and the error rate of the signal state, respectively.

When QKD coexists with classical signals, the noise generated by the classical signals primarily impacts the background noise count rate ($Y_{0}$) of the QKD system and, consequently, the secure key rate. $Y_{0}$ can be referred to as the total noise photon probability and is expressed as shown in this research:

(11) $$Y_{0}=2p_{dark}+p_{SpRS}+p_{FWM}.$$

The SpRS and FWM noise count rates can be calculated as follows:

(12) $$\left\{\begin{array}{c} p_{SpRS}=\frac{P_{SpRS}\times \Delta f\times \eta_{spd}\times \tau_{gate}}{h\times f},\mathrm{for}\;\mathrm{SpRS}\;\mathrm{noise},\\ p_{FWM}=\frac{P_{FWM}\times \eta_{spd}\times \tau_{gate}}{h\times f},\mathrm{for}\;\mathrm{FWM}\;\mathrm{noise}, \end{array}\right.$$

where $\eta_{spd}$ and $\tau_{gate}$ denote the detector efficiency and gating width of the single photon detector (SPD), respectively, while Δf signifies the receiving bandwidth of the quantum channel, *h* is Planck’s constant (6.63 × $10^{-34}\;J$· s) and *f* is the frequency of the quantum signal. Finally, $p_{SpRS}$ and $p_{FWM}$ are dimensionless.

SpRS noise is distributed over a wide range of wavelengths, and the receiver bandwidth needs to be considered. FWM noise is considered monochromatic, and thus the receiver bandwidth is not considered.

## 3. Simulation Set-Up and Results

To assess the noise power and QKD performance with different schemes over HCF, MCF and SCF, simulation analyses were conducted. The simulation parameters are detailed in Table 1.

### 3.1. Noise Analysis

**Figure 3:**
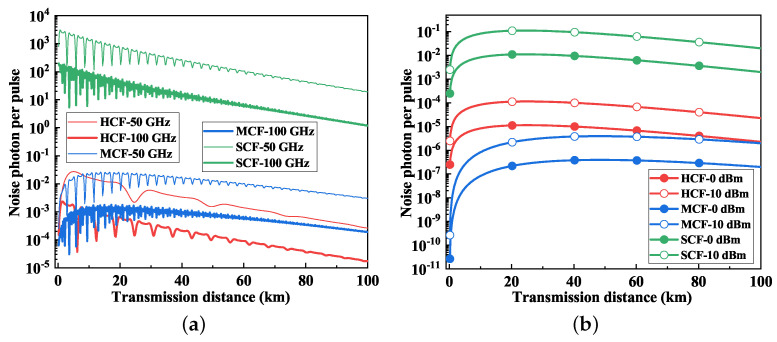
The noise photon counts vs. the fiber distance. (**a**) FWM noise. (**b**) SpRS noise.

Figure 3 shows the relationship between the photon count and the transmission distance of FWM noise and SpRS noise in the quantum channel. The power of the classical signal per channel was 10 dBm. In Figure 3a, the photon count of the FWM noise under varying channel spacings is analyzed over HCF, MCF and SCF. The spacings between the classical channels, as well as between the classical channel and quantum channel, were the same. Increasing the channel spacing could suppress the FWM noise in the quantum channel for different fiber types. The photon count of the FWM noise in SCF was notably higher than in HCF and MCF. Within 5 km, the FWM noise photon count in HCF surpassed that of MCF, but this trend reversed beyond 5 km. As the transmission distance grew, the FWM photon count in HCF became lower than in MCF. This discrepancy can be attributed to the lower nonlinear coefficient of HCF, whereas the nonlinear coefficient of MCF aligned closely with that of SCF, and the isolation between cores in MCF attenuated the FWM effect. Additionally, the power fluctuation characteristics of HCF differed from those of MCF and SCF due to their distinct fiber dispersion properties. In addition to the nonlinear coefficients which affect the power of the FWM noise, dispersion also plays a significant role in the power of FWM and causes fluctuations with the distance [36]. In the simulation, the dispersion of HCF was 2 ps/(nm·km), and the fiber dispersion of MCF and SCF was 17 ps/(nm·km) [15].

Figure 3b shows the relationship between the count of SpRS noise photons and the transmission distance. The count of SpRS noise photons in the quantum channel initially rose and then declined. One significant observation is that the photon count of the SpRS noise in HCF surpassed that in MCF, which is crucial when considering the simultaneous transmission of classical and quantum signals.

**Figure 4:**
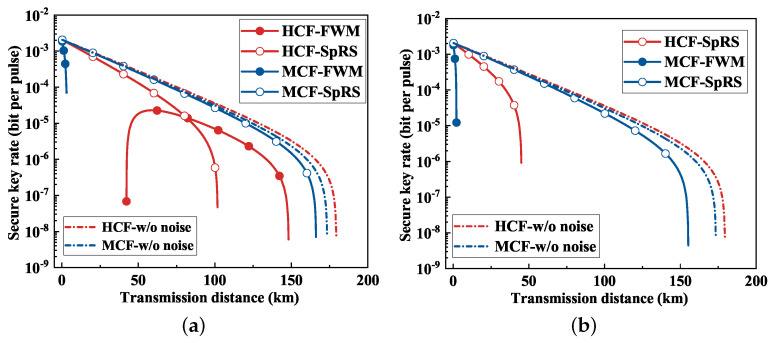
Impact of noise on QKD performance. (**a**) Four classical channels. (**b**) Eight classical channels. (w/o = without).

Figure 4 illustrates the influence of FWM noise and SpRS noise on the QKD performance. The power of the classical signal per channel was maintained at 10 dBm.

Regarding FWM noise, in Figure 4a, where there are four classical channels, FWM noise in the HCF emerges as the predominant noise source, significantly compromising QKD performance. Within a 50 km transmission distance, the FWM noise in the quantum channels became excessively pronounced, which rendered the QKD system non-operational. Nevertheless, with increasing transmission distances exceeding 50 km, the FWM noise diminished, thereby facilitating secure key rates within the range from 50 km to 150 km. Beyond 150 km, the incapability of the QKD system to function can be attributed to substantial fiber attenuation. In Figure 4b, with eight classical channels, the QKD system with FWM noise cannot work across all transmission distances in the HCF. Despite FWM noise dominating in the MCF scenario, the system could sustain transmission up to at least 2 km.

For SpRS noise, the influence of SpRS noise differed between HCF and MCF. Specifically, SpRS noise exerted a more pronounced impact on QKD performance in HCF compared with MCF. With the utilization of four classical channels in Figure 4a, MCF showed a transmission distance extended by 50 km relative to HCF. Furthermore, when employing eight classical channels in Figure 4b, this transmission distance in MCF expanded to over 100 km. The secure transmission distance achievable for QKD systems, free from noise interference, approached approximately 175 km in both HCF and MCF. Consequently, the presence of noise in HCF and MCF significantly impaired the performance of QKD systems, emphasizing the critical need for effective noise suppression measures.

### 3.2. Scheme Analysis

**Figure 5:**
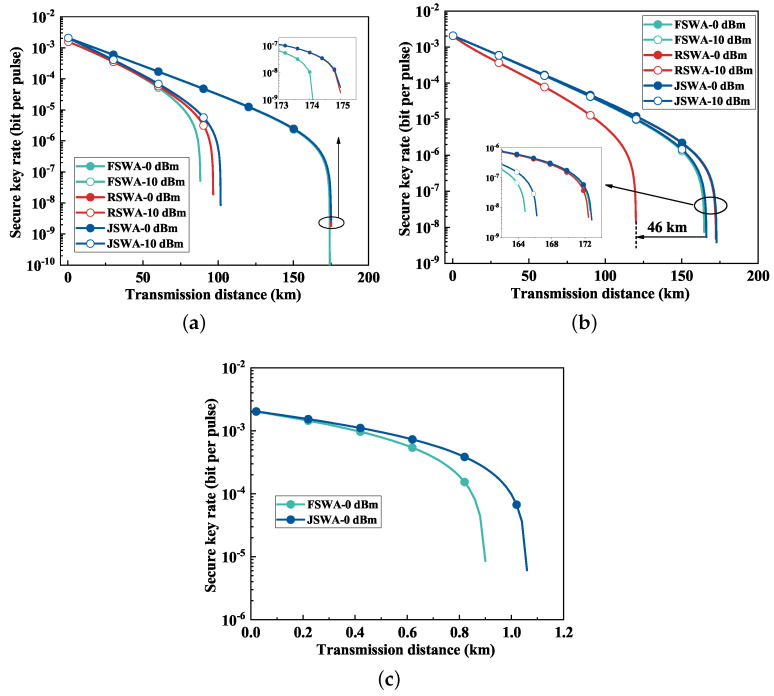
The relationship between a secure key rate and the transmission distance with different schemes. (**a**) HCF. (**b**) MCF. (**c**) SCF.

In this section, the allocation of classical channels and quantum channels is shown in Table 2. CCh denotes the classical channel, and QCh represents the quantum channel. Figure 5 presents the evaluation of various schemes across different fiber types. Figure 5a specifically focuses on the QKD performance in HCF. When the classical signal power per channel was set to 0 dBm, the QKD secure transmission distances achieved with the FSWA, RSWA and JSWA schemes could extend up to 175 km, attributed to minimal noise interference. The JSWA scheme showed a distinct advantage with a classical signal power per channel of 10 dBm, achieving a secure transmission distance of 100 km. Although the FSWA and RSWA schemes are vulnerable to SpRS noise and FWM noise, respectively, they still achieved a transmission distance of 80 km.

The performance evaluation of the proposed scheme in MCF is shown in Figure 5b. With classical power levels ranging from 0 dBm to 10 dBm, the transmission distances achieved by the JSWA and RSWA schemes exhibited a negligible reduction of less than 10 km. Notably, the secure transmission distance associated with the RSWA scheme was reduced by 46 km compared with that of the JSWA scheme. This disparity primarily stemmed from the heightened FWM noise at higher classical power levels.

In SCF, the profound noise influence from classical signals restricted the maximum transmission distance to merely about 1 km at a classical signal power per channel of 0 dBm, as shown in Figure 5c. Comparatively, the secure transmission distance under the JSWA scheme marginally surpassed that of the FSWA scheme. Conversely, the QKD system could not work under the RSWA scheme, primarily attributed to the pervasive impact of FWM noise.

**Figure 6:**
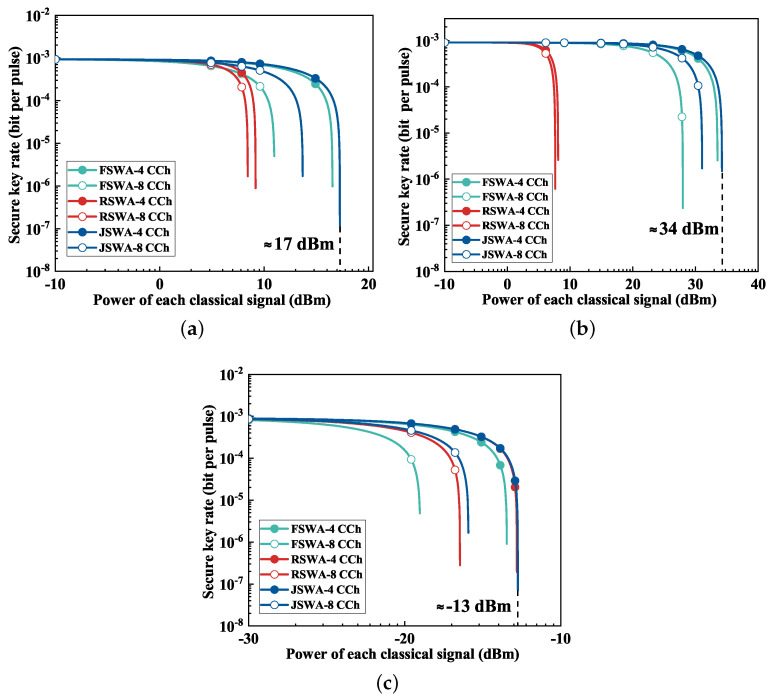
The relationship between a secure key rate and classical power with different schemes. (**a**) HCF. (**b**) MCF. (**c**) SCF.

Figure 6 illustrates the coexistence capabilities of QKD with high-power classical communication across 20 km of different fiber types. In Figure 6a, focusing on HCF, the RSWA scheme indicates limited tolerance to classical power, capped at 10 dBm, which can be attributed to ineffective suppression of FWM noise. The FSWA scheme could further enhance its classical signal power tolerance due to the effective suppression of FWM noise. Furthermore, the JSWA scheme exhibited a higher classical power tolerance, reaching approximately 17 dBm per channel and outperforming the RSWA scheme on both four and eight classical channels.

Figure 6b presents the QKD performance in MCF. Despite the RSWA scheme’s classical power tolerance remaining capped at 10 dBm across four and eight classical channels, the tolerable power for JSWA schemes approached or even surpassed 30 dBm under eight classical channels. Compared with the classical power tolerance in HCF, this represents an advantage of over 10 dB, which can be attributed to the effective suppression of FWM noise. This improvement significantly facilitates the coexistence of QKD with large-capacity or high-power classical communication systems.

Figure 6c presents the QKD performance in SCF. The FSWA scheme showed suboptimal performance with both four and eight classical channels, with a maximum tolerable classical signal power not exceeding −10 dBm. This limitation is primarily attributed to SpRS emerging as the dominant noise source at low classical power levels.

**Figure 7:**
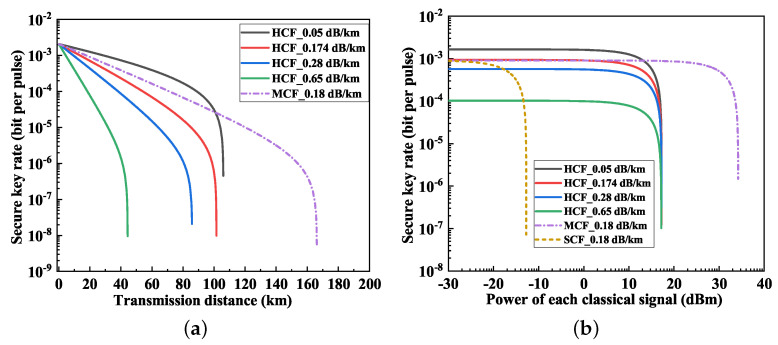
QKD performance with different fiber attenuation levels. (**a**) Secure key rate vs. transmission distance. (**b**) Secure key rate vs. classical power.

The theoretical attenuation limit in HCF was approximately 0.05 dB/km [14,18]. To investigate the impact of fiber attenuation on QKD, we examined the QKD performance with the JSWA scheme in HCF under varying attenuation levels, as depicted in Figure 7. In Figure 7a, the relationship between the secure key rate and transmission distance is illustrated. The classical power per channel was 10 dBm. Initially, the secure key rates across the HCF variants with different attenuations were close, determined by the inherent parameters of the QKD system. However, as the transmission distance extended, the secure key rate in high-attenuation HCF experienced a precipitous decline, consequently reducing the secure transmission distance. Although MCF had an attenuation rate of 0.18 dB/km, its secure transmission distance still surpassed that of HCF with 0.05 dB/km. Thus, MCF emerged as a more suitable candidate for long-distance transmission of QKD and classical communications, attributed to the spatial isolation provided by its multi-core structure.

Figure 7b illustrates the relationship between the secure key rate and the power of the classical signal using the JSWA scheme. The transmission distance was 20 km. In MCF, the power of the classical signal which could be accommodated surpassed 30 dBm. Conversely, in HCF, varying levels of attenuation impacted the secure key rate, limiting the tolerable power of the classical signal to 17 dBm due to the consistent SpRS noise observed.

**Figure 8:**
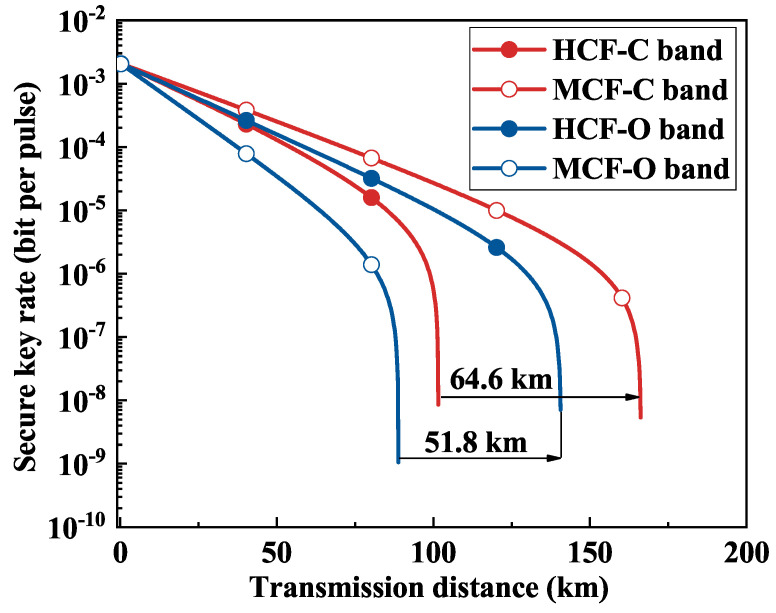
QKD performance analysis in different bands.

In order to assess the performance of QKD over different bands, we conducted an analysis of QKD performance in the C-band and O-band scenarios, as shown in Figure 8, with classical signals confined to the C-band. The classical power per channel was 10 dBm. As previously detailed, in the C-band scenario, QKD experienced attenuated effects from the SpRS noise in MCF, resulting in an extension of the secure transmission distance by approximately 64.6 km compared with HCF.

In the O-band scenario, the fiber attenuation in HCF was 0.22 dB/km and 0.35 dB/km (reference SCF attenuation level) in MCF [17]. The secure transmission distance of QKD in HCF was extended by about 51.8 km compared with MCF. This improvement can be attributed to noise reduction and the inherent advantage of lower attenuation, thus extending the secure transmission distance.

### 3.3. Results Discussion

The RSWA scheme and FSWA scheme effectively mitigated a portion of the noise, thereby enhancing the performance of QKD. Furthermore, the JSWA scheme employs a channel interleaving strategy, enabling complete suppression of FWM noise and minimizing SpRS noise. As a result, the JSWA scheme indicated superior performance compared with the RSWA scheme and FSWA scheme. However, in engineering applications, the RSWA and FSWA schemes demand lower isolation requirements compared with the JSWA scheme, making them easier to implement.

The secure transmission distance for QKD and the classical signals in HCF and MCF surpassed that of SCF. The advent of HCF and MCF presents opportunities for integrating QKD with classical communications. In low-noise environments, such as when classical signal power is low or when quantum and classical signals operate in different bands, QKD coexistence with classical signals in HCF indicated superior performance compared with MCF, primarily due to reduced fiber attenuation. As HCF approaches its theoretical attenuation limit, this advantage is expected to become even more pronounced.

In high-noise scenarios, such as when there is high classical signal power or when classical and quantum signals operate in the same band, the situation is reversed. The coexistence of classical and quantum signals in MCF can offer longer transmission distances or higher secure key rates compared with HCF. Although noise from MCF effects is comparable to that of SCF, spatial division multiplexing in MCF facilitates isolation between classical and quantum signals, thereby reducing noise interference. In this study, we considered homogeneous MCF configurations, and the use of heterogeneous MCF could further enhance the performance of QKD.

As new types of optical fibers, HCF and MCF are highly regarded in the field of classical and quantum signal coexistence. In addition, with the advantages of low attenuation, minimal noise and low latency, HCF is well suited for high-power communication scenarios [46] and edge data centers [47]. For MCF, characterized by its large capacity and high spatial efficiency, it is ideal for long-distance, high-capacity communications and compact space applications, such as undersea data transmission [48] and inter-data center connections [49]. With continued advancements leading to further reductions in HCF attenuation and MCF fan-in/fan-out loss, the advantages and applications of these fibers are expected to expand significantly.

This study primarily employed simulation analysis to evaluate the proposed JSWA scheme. Future experimental verification is essential for its optimization and practical implementation. There may be two main directions for future experimental verification. (1) Regarding the feasibility of the proposed scheme in practical experiments, because while the proposed scheme showed excellent performance in the simulation analysis, some factors in the simulation were idealized. Experiments often consider more practical and real-world variables. Therefore, it is crucial to evaluate the performance of the proposed scheme under experimental conditions. (2) For the tolerance of QKD to high-power classical communication, using the JSWA scheme, QKD can coexist with classical power levels of 17 dBm per channel, and the MCF can reach up to 30 dBm. However, additional factors may be introduced in an experimental platform. It is essential to further evaluate the feasibility of QKD operating alongside high-power classical communication while ensuring the QKD system functions correctly.

The experimental challenges may include the following. (1) High link loss, as the commercial QKD system typically tolerates link loss up to 16 dB [19,30]. While HCF can achieve low loss in theoretical or laboratory settings, its loss remains higher than that of SCF in mass production due to the immature manufacturing process. Considering the additional loss from devices, this presents a significant challenge for the QKD system. Hence, experimental evaluations may only be feasible with short-distance HCF. (2) Concerning non-tunable QKD wavelengths, the JSWA scheme involves multiple quantum wavelengths. However, the QKD system’s wavelength is usually fixed, complicating multi-quantum wavelength performance evaluation in the JSWA scheme. Initially, experimental evaluations can be conducted by a limited number of fixed-wavelength QKD devices. (3) Finally, as for channel crosstalk, the JSWA scheme interleaves classical and quantum channels with small spacing. The crosstalk noise from classical signals, due to inadequate device isolation, may affect QKD performance. Therefore, high-isolation devices are necessary for further experiments.

## 4. Conclusions

In this paper, we conducted an analysis of SpRS noise and FWM noise across HCF, MCF and SCF. We proposed noise-suppressed wavelength allocation schemes for QKD to facilitate the coexistence of classical communication within these fiber types. Our findings indicate that the JSWA scheme effectively suppresses FWM noise, mitigates SpRS noise and substantially extends the secure transmission distance across HCF, MCF and SCF. Compared with SCF, HCF demonstrated a significant reduction in noise’s impact on QKD. Furthermore, MCF offers enhanced noise immunity due to spatial isolation, allowing for extended transmission distances of QKD in the C-band and the capability to tolerate classical communications at power levels exceeding 30 dBm.

## Figures and Tables

**Table 1:** Simulation parameters.

Parameter	Value
Fiber attenuation (HCF, MCF and SCF)	0.174, 0.18 and 0.18 dB/km
Coupling coefficient of MCF	$10^{-6}$/km
Average number of photons per signal pulse	0.4
Error correction efficiency	1.15
Detector efficiency	20%
Detection probability of dark counts	$10^{-6}$
Detection gate width	1 ns

**Table 2:** Channel allocation under schemes.

Schemes	4 CCh	8 CCh
FSWA	CCh: [193.6, 0.1, 193.9] THz; QCh: 194.3 THz.	CCh: [193.6, 0.1, 194.3] THz; QCh: 195.1 THz.
RSWA	CCh: [193.6, 0.1, 193.9] THz; QCh: 194.0 THz.	CCh: [193.6, 0.1, 194.3] THz; QCh: 194.4 THz.
JSWA	CCh: [193.6, 0.1, 193.9] THz; QCh: 194.05 THz.	CCh: [193.6, 0.1, 194.3] THz; QCh: 194.4 THz.

## Data Availability

The data presented in this study are available on request from the corresponding author.
